# QTL analysis reveals quantitative resistant loci for *Phytophthora infestans* and *Tecia solanivora* in tetraploid potato (*Solanum tuberosum* L.)

**DOI:** 10.1371/journal.pone.0199716

**Published:** 2018-07-06

**Authors:** Juan David Santa, Jhon Berdugo-Cely, Liliana Cely-Pardo, Mauricio Soto-Suárez, Teresa Mosquera, Carlos H. Galeano M.

**Affiliations:** 1 Corporación Colombiana de Investigación Agropecuaria (AGROSAVIA), C.I. Tibaitatá, Cundinamarca, Colombia; 2 Faculty of Agricultural Sciences, Universidad Nacional de Colombia, sede Bogotá, Colombia; 3 Corporación Colombiana de Investigación Agropecuaria (AGROSAVIA), C.I. Palmira, Valle del Cauca, Colombia; USDA/ARS, UNITED STATES

## Abstract

Late blight and Guatemalan potato tuber moth caused by *Phytophthora infestans* and *Tecia solanivora*, respectively, are major phytosanitary problems on potato crops in Colombia and Ecuador. Hence, the development of resistant cultivars is an alternative for their control. However, breeding initiatives for durable resistance using molecular tools are limited due to the genome complexity and high heterozygosity in autotetraploid potatoes. To contribute to a better understanding of the genetic basis underlying the resistance to *P*. *infestans* and *T*. *solanivora* in potato, the aim of this study was to identify QTLs for resistance to *P*. *infestans* and *T*. *solanivora* using a F1 tetraploid potato segregant population for both traits. Ninety-four individuals comprised this population. Parent genotypes and their progeny were genotyped using SOLCAP 12K potato array. Forty-five percent of the markers were polymorphic. A genetic linkage map was built with a length of 968.4 cM and 1,287 SNPs showing good distribution across the genome. Severity and incidence were evaluated in two crop cycles for two years. QTL analysis revealed six QTLs linked to *P*. *infestans*, four of these related to previous QTLs reported, and two novel QTLs (qrAUDPC-3 and qrAUDPC-8). Fifteen QTLs were linked to *T*. *solanivora*, being qIPC-6 and qOPA-6.1, and qIPC-10 and qIPC-10.1 stable in two different trials. This study is one of the first to identify QTLs for *T*. *solanivora*. As the population employed is a breeding population, results will contribute significantly to breeding programs to select resistant plant material, especially in countries where *P*. *infestans* and *T*. *solanivora* limit potato production.

## Introduction

Potato (*Solanum tuberosum* L.) is the third most consumed food crop worldwide after rice and wheat (FAOSTAT 2017). However, late blight caused by *Phythopthora infestans* (Mont) de Bary [1] and the Guatemalan potato tuber moth (*Tecia solanivora* (Lepidoptera: Gelechiidae)), are the main phytosanitary problems for potato in Colombia and Ecuador, generating up to 40% in annual losses, and even severe epidemics have been reported [2–4]. Moreover, these phytosanitary problems may even reach a 100% of loss in some cases under field conditions and during storage, as some potato varieties used in Colombia are susceptible to Guatemalan potato moth (GPTB) and late blight (LB) [5]. Hence, pests and diseases management requires an extremely high input of pesticides [6–8]. In this sense, it is relevant to look for resistance sources for these phytosanitary problems and especially for *T*. *solanivora*, for which available information is limited.

The Andigenum group in potatoes includes diploid, triploid and tetraploid individuals collected from high Andean regions [9]. Moreover, autotetraploid potatoes have tetrasomic inheritance (2n = 4x = 48), high heterozygosity (> 0.8), self-incompatibility [10] and high genetic diversity [11]. Thus, the potato genome has structural complexity that limits the analysis of quantitative trait loci (QTL) [12,13] and the potential use of marker assisted selection (MAS) [14]. Additionally, the Andigenum group is considered as an important source of resistance genes [15], with a broad genetic base that allows the induction of positive heterosis for potato breeding [16].

The Colombian germplasm bank (Banco de Germoplasma de la Nación Colombiana) maintains 675 tetraploid accessions inside the Colombian Central Collection (CCC) of potato, which is considered as one of the most diverse potato collections in the world. In 2017, the CCC was genotyped and a preliminary analysis reported single nucleotide polymorphism (SNP) associated to morphological traits [11]. However, studies to evaluate the loci involved on agronomic traits such as yield, pests and diseases resistance, abiotic tolerance, and tuber quality should be evaluated.

Authors as Ramakrishnan *et al*. [17] compiled the information generated on potato molecular breeding and reported 24 markers linked to different major resistance genes (*R* genes), identified mainly in diploid populations. Most of the *R* genes were derived from the wild potato species *Solanum demisum* Lindl., containing nucleotide-binding (NB) and leucine-rich repeat (LRR) domains, which leads to programmed cell death through hypersensitive response (HR) [18]. This monogenic resistance exerts a specific selection pressure to the pathogen, forcing the emergence and spread of new pathogen races [19]. In this sense, for resistance to LB a consensus map clustering 144 previous reported QTLs reveled 24 meta-QTLs involved on quantitative resistance [20,21].

In autotetraploid potato populations the use of SNP markers has facilitated the analysis of tetrasomic inheritance in autotetraploid species, where five different types of allelic dosage, and 13 types of expected segregation from nulliplex (AAAA) to quadruplex (BBBB) genotypes can be identified [22–23]. Advances in genotyping platforms such as the Infinium Potato Array [24,25] and the sequenced potato genome [26], have allowed a high-density genotyping on tetraploid populations. These technologies have facilitated QTL analysis to identify markers related to agronomic traits as virus resistance [27], tuber quality [28] and resistance to *P*. *infestans* [1,14,29]. Furthermore, the development of statistical models have allowed the incorporation of allelic dosage information, increasing the probability to detect recombination frequencies between loci for QTL mapping on autopolyploid species [22,23,30–33].

Despite these advances, limited studies related to understanding the genetic underlying potato pest resistance has been reported, and specifically the resistance to *T*. *solanivora* has poorly been studied and understood. Recently, as part of the project “Aprovechamiento de los recursos genéticos para la valorización de sistemas productivos sostenibles de papa” [Use of potato genetic resources for value generation in sustainable productive systems] developed by AGROSAVIA, *T*. *solanivora* resistance sources within the Andigenum population from the CCC of potato have been evaluated. From this study, we selected and crossed five genotypes with partial resistance, with commercial cultivars generating 34 full-sib F1 families. Among these, the population RN × 2384 developed from the crossing between Roja Nariño as the LB resistance source, and BGVCOL 15062384 as the moth resistance source, were used in this study. According to the aforementioned, the aim of this study was to develop a linkage map and identify QTLs for resistance to *T*. *solanivora* and *P*. *infestans* in the RN x 2384 biparental population.

## Materials and methods

### Plant material

We employed the F1 biparental population (RN × 2384) comprised by 94 full siblings resulting from a crossing between cultivar Roja Nariño (RN) and the landrace BGVCOL 15062384. Roja Nariño is a commercial cultivar with quantitative resistance to *P*. *infestans* and susceptible to *T*. *solanivora* [5], whereas BGVCOL 15062384 is a native Andean variety maintained in the CCC of potato which is susceptible to *P*. *infestans*, and was selected for its potential resistance against *T*. *solanivora* under controlled conditions, according to Cely and Barreto (unpublished data).

#### Field assay and experimental design

The segregating population was evaluated in two crop cycles during 2015 and 2016 in Tibaitatá Research Center of AGROSAVIA, located in the municipality of Mosquera in Colombia. The research center is located at 4° 68' 94” N and 74° 20' 49” W, and at 2,550 m a.s.l. Field trials included an experimental unit of 12 plants per genotype with a spacing between plants of 0.40 m and 1 m between rows. Trials were established in July 2015 under an augmented block design with six checks and six blocks, and in June 2016 under a randomized complete block design with three replicates. The LB and GPTM damage was evaluated in both years. Pests and diseases were managed with a standard chemical control until 20 days after emergence (dae) and then, applications were suspended. As a resistant and a susceptible control, the cultivars Pastusa Suprema and Diacol Capiro were used, respectively.

#### Evaluation of resistance to *Phytophthora infestans*

The *P*. *infestans* severity-based disease progress under field conditions was scored during five observations every 15 days starting from the 30^th^ dae for the trials in 2015 and 2016, under natural *P*. *infestans* infestation conditions. Percentage of the disease-infected area compared to the whole plant was estimated visually in four plants in each experimental unit [34]. The average of affected leaf area value was used to calculate the area under the disease progress curve (AUDPC):

$$AUDPC=\sum_{i:1}^{n}\left(\frac{Xi+1+Xi}{2})(ti+1-ti\right)$$ (1)

Where, *Xi* is the percentage of tissue affected at observation *i*; *ti* is observation days, and *i* is the number of observations.

#### Evaluation of resistance to *Tecia solanivora*

We evaluated the resistance to *T*. *solanivora* in the field during 2015 and 2016 as well as under storage conditions in 2017. For field conditions, we evaluated weekly the adult population density of *T*. *solanivora* using a sexual pheromone trap [35]. Pest damage incidence at harvest (IPC) was calculated as a ratio of the number of tubers with damage over the total number of tubers. For storage conditions in 2017 (15 ± 5°C, 74 ± 20% relative humidity (RH)), moth incidence (IPA), moth severity (SPA) and the number of moth larvae outflow holes (OPA) were evaluated, employing in this case, entomological cages of 6.25 m^2^ under a randomized complete block design. Infestations were carried out using 50 adult couples of Guatemalan moths released weekly for five weeks. Subsequently, IPA and OPA values were scored four weeks after the last infestation, as mentioned above. SPA was evaluated cutting the affected tuber into quarters and each quarter was visually evaluated according to the damage scale published by Arias *et al*. [36]. In addition, means of each variable were adjusted according to differences between blocks. Phenotypic statistics data and normality test (Shapiro-Wilk, Kurtosis, Skewness) were carried out using the R software version 3.4.1 [37].

#### Genotyping

Young leaf samples of eight weeks after emergence were used for DNA extraction. DNA was extracted using the DNeasy Plant Mini kit (Qiagen, Germantown, MD, U.S.A.) according to instructions from the manufacturer. DNA quality and concentration was checked by spectrophotometry in a Nanodrop (Nanodrop Technologies, Montchanin, DE, U.S.A.), and visualized in a 1% agarose gel stained with SYBR Safe (0.5 μm mL^-1^). Samples were diluted to a final concentration of 50 ng μL^-1^. Moreover, genotyping was carried out using the Infinium (Illumina®) platform with SolCAP potato 12808 SNP array (GeneSeek, San Diego, CA, U.S.A.). SNP allele fluorescence intensities obtained from Genome Studio software version 2.0 (Illumina, San Diego, CA, U.S.A.) were used to assign five possible tetraploid genotypes (AAAA, AAAB, AABB, ABBB, BBBB) in each sample using the saveMarkerModels option included in the fitTetra package [30] using the statistical R software [37].

#### Genetic linkage map construction

A linkage map was developed using TetraploidSNPMap software section SNP (TPM, BIOS) [33]. SNP markers with significance values of the chi-square goodness-of-fit statistic that were lower than 0.001 for SNP simplex and 0.01 for SNP duplex and other types of segregation distortion were discarded. Furthermore, SNPs in linkage cluster analysis groups were mapped [38] and ordered according to the chromosome in the physical map (PGSC v4.03) [26]. Additionally, the linkage phase of each marker and an analysis between pairs of markers were estimated by two-point analysis (TPA). A multidimensional scaling analysis (MDS) was carried out according to the methodology reported by Preedy and Hackett [32], deleting markers placed outside the calculated curve. Then, recombination frequencies (RF) were calculated and mapped with a RF < 0.5 and transformed to relative distances in centi-Morgans (cM) using the Kosambi function [39]. Parent phase was inferred using the dosage of simplex markers of the population in each homologous chromosome. A graphical representation of the linkage map was carried out using MapChart 2.3 [40].

#### *Phytophthora infestans* and *Tecia solanivora* resistance QTL mapping

We adjusted the average of each variable according to the experimental design analyzed with the genotyping matrix using TetraploidSNPMap software section SNP-QTL (TPM, BIOS) [33]. A model of additive effects for each homologous chromosome was used for QTL mapping. The statistical significance of the QTL was based on 1,000 permutations with a 95% confidence interval. Furthermore, the percentage of explained variance by each QTL and coefficient of determination *R*^2^ was calculated for each variable: severity of LB in the field for each year (rAUDPC), incidence of GPTB in the field per year (IPC), incidence of GPTB under storage conditions (IPA), severity of GPTB under storage conditions, and number of output holes in storage (OPA).

Nonetheless, the most likely QTL position was calculated based on simple models according to the means of the phenotypic values using the Schwarz information criterion (SIC) [41]. Models tested were simplex, duplex as codominant variant, duplex as codominant factor, allele dominant duplex, double simplex as codominant variant, codominant factor double simplex, and dominant double simplex [33]. The model with the lowest SIC and a highest *R*^2^ values were selected following the methodology proposed by Hackett *et al*. [23].

## Results and discussion

### Phenotypic evaluation

Phenotypic data follow a normal distribution (Kurtosis < 1, Skewness close to 0 and Shapiro-Wilk *p*-value > 0.05), with exception of two traits: IPC in 2016 and OPA in 2017 (Table 1).

**Table 1:** Resistance evaluation to *Phytophthora infestans* and *Tecia solanivora* in the potato segregating tetraploid population RN × 2384.

Trait*	Year	Parents**			F1 progeny								
		Roja Nariño	BGVCOL 15062384										
				Mean	SD	Min	Max	SD	SE	Var	Kurtosis	Skewness	SW *p-value*
**rAUDPC**	2015	-	-	0.16	0.04	0.08	0.26	0.037	0.003	0.0014	-0.22	0.20	0.578
**rAUDPC**	2016	0.11	0.60	0.43	0.12	0.16	0.72	0.118	0.012	0.0140	-0.14	-0.16	0.7215
**IPC (%)**	2015	-	-	27.38	17.03	0.70	73.20	17.13	1.796	239.76	-0.08	0.72	0.0008
**IPC (%)**	2016	20.98	2.41	5.07	4.88	0.00	33.33	4.93	0.526	24.382	11.22	2.76	7.39 x 10^−11^
**IPA (%)**	2017	34.54	15.78	29.93	13.88	5.07	66.72	13.87	1.487	192.59	-0.68	0.38	0.05479
**SPA (%)**	2017	52.08	24.71	51.58	5.07	24.38	81.84	12.04	1.291	145.08	-0.45	-0.01	0.8324
**OPA (holes)**	2017	2.0	0.97	1.92	66.72	0.89	4.64	1.043	0.111	1.089	19.48	3.56	6.05 X 10^−12^

* Values are presented in terms of severity mean values, maximum (Max) and minimum values (Min), standard deviation (SD), standard error (SE), variance (Var), Kurtosis, Skewness and Shapiro-Wilk *p-value* (SW *p-value*). *P*. *infestans* relative area under the disease progress curve (rAUDPC); *T*. *solanivora* field incidence (IPC), *T*. *solanivora* incidence under storage (IPA), *T*. *solanivora* severity under storage (SPA); and number of outflow holes in storage (OPA).

** Parents were only evaluated in 2016 and 2017.

Field condition results were directly related to rainfall. Hence, due to the low precipitation occurred in year 2015 (178 mm), the severity of *P*. *infestans* showed rAUDPC values from 0.08 to 0.26, with an average of 0.16. However, due to an increase in rainfall up to 425 mm occurred in the flowering stage during 2016, severity decreased ranging between 0.16 and 0.72 with an average of 0.43 (Table 1 and S1 Table). These environmental contrasting conditions were very important for the evaluation of the resistance to GPTB and LB.

**Fig 1:**
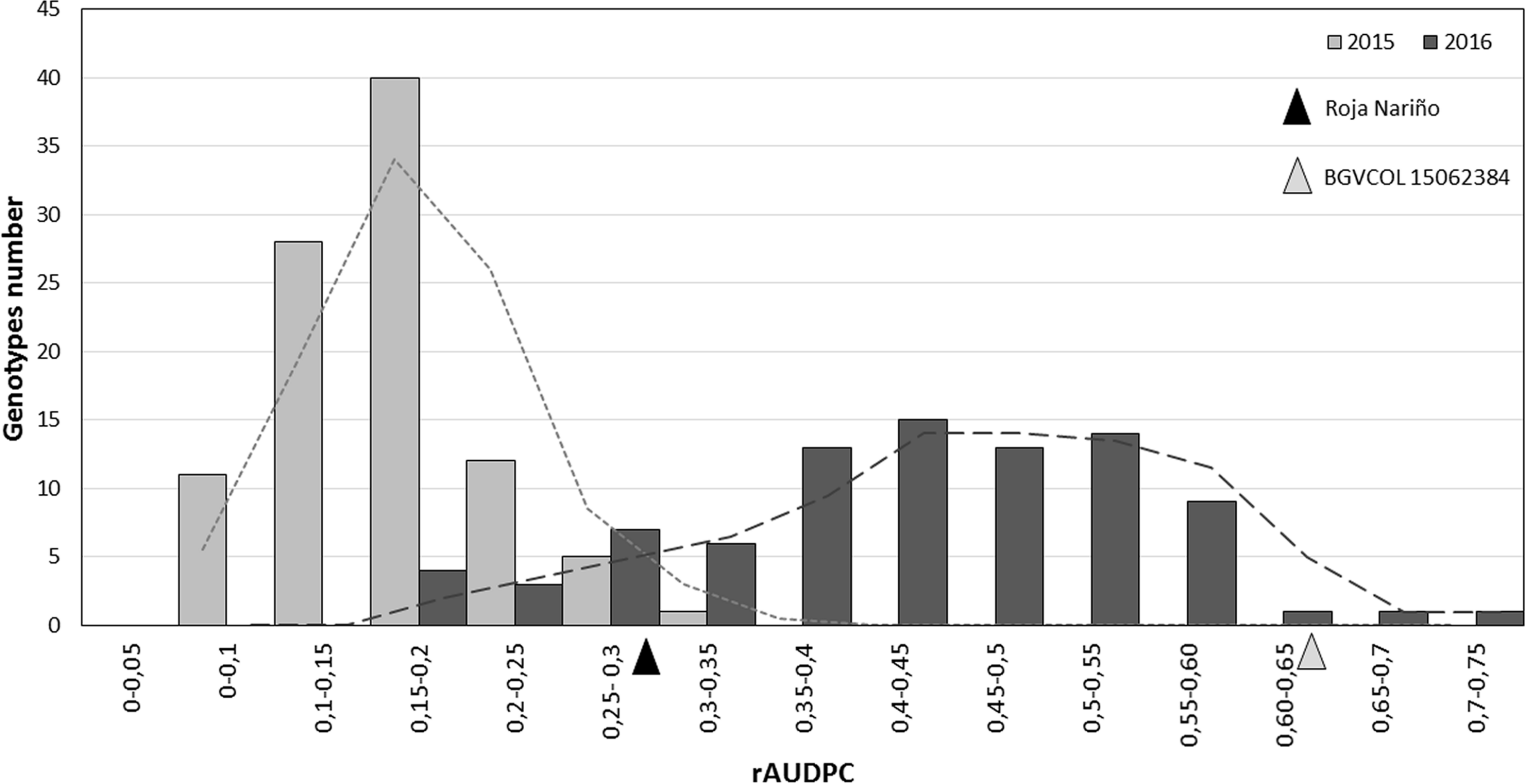
Histogram of severity of *Phythopthora infestans*. The x-axis shows the relative area under the disease progress curve (rAUDPC) and the y-axis the number of genotypes can be observed. The dash lines indicate the average trend for each year. The black and grey triangles indicate the phenotypic parent values of Roja Nariño and BGVCOL 15062384 in 2016, respectively.

Despite different weather effects occurred in each year (S1 Fig), rAUDPC and IPC traits in 2015, RAUDPC in 2016, and IPA and SPA in 2017, exhibited normal distribution showing polygenetic effects associated with LB and GPTM resistance. Specifically, in 2016 a transgressive segregation was obtained with more resistant and susceptible genotypes to *P*. *infestans* than their parents (Fig 1). Similarly, Costanzo *et al*. [42] found 13 genotypes with lower AUDPC values than the resistant parent BD172-1 (*Solanum stenotomum* Juz. & Bukasov) using a two-parent F1 diploid population of *Solanum phureja* Juz. et Buk × *S*. *stenotomum* including 230 full-sibs. In our study, high precipitation and high relative humidity occurred in 2016 comprising important factors to increase the *P*. *infestans* epidemic in the field. Sporangia are particularly formed in a humidity of 90% to 100%, and then water on the leaf surface allowed the production, release and dispersion of zoospores [4], while sporangia dispersion depends on wind speed [43].

**Fig 2:**
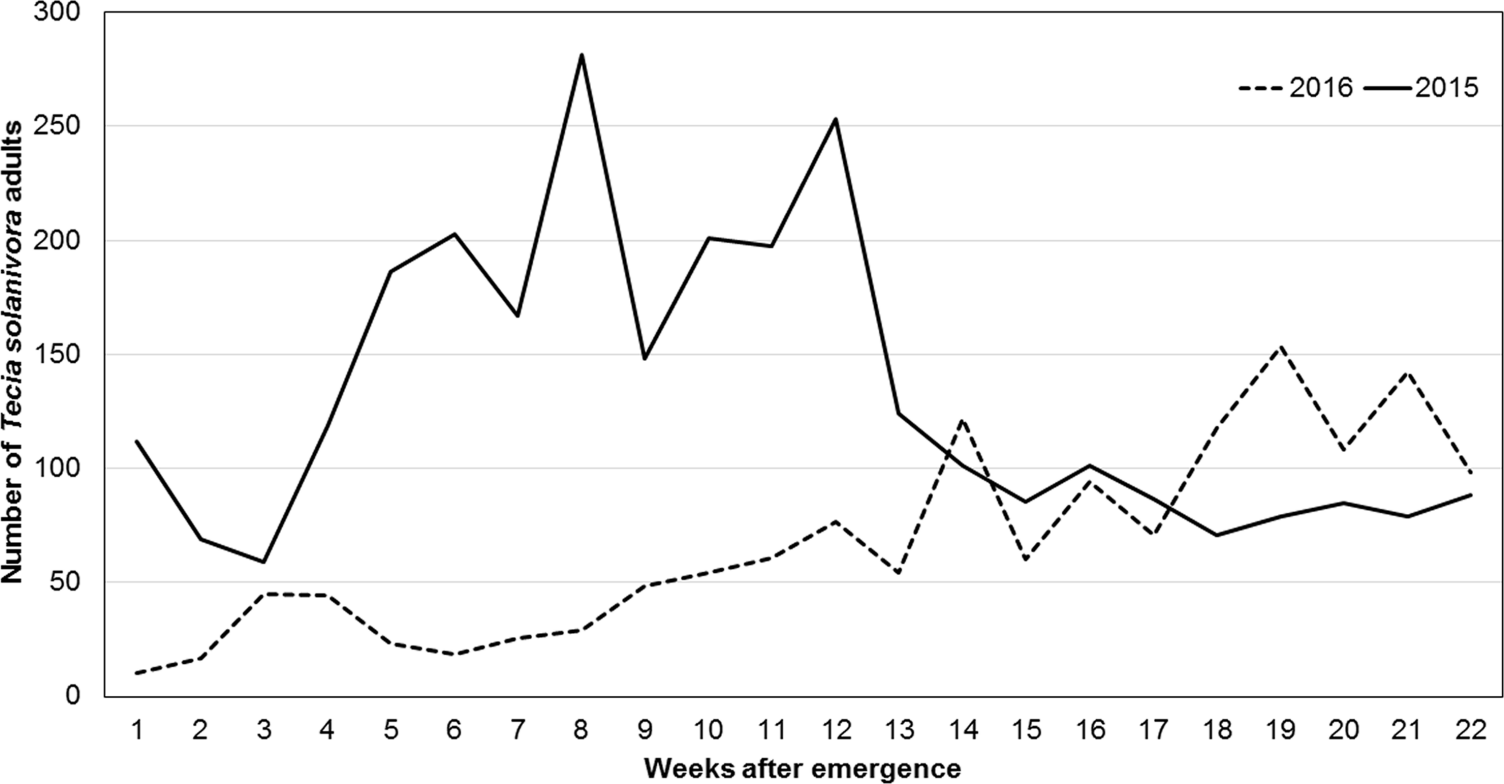
Population screening of *Tecia solanivora*. Fluctuation of adult male populations of *Tecia solanivora* during the field trials for years 2015 and 2016. Evaluations were conducted weekly using a sexual pheromone trap during two crop cycles in consecutive years 2015 (continuous line) and 2016 (dotted line).

**Fig 3:**
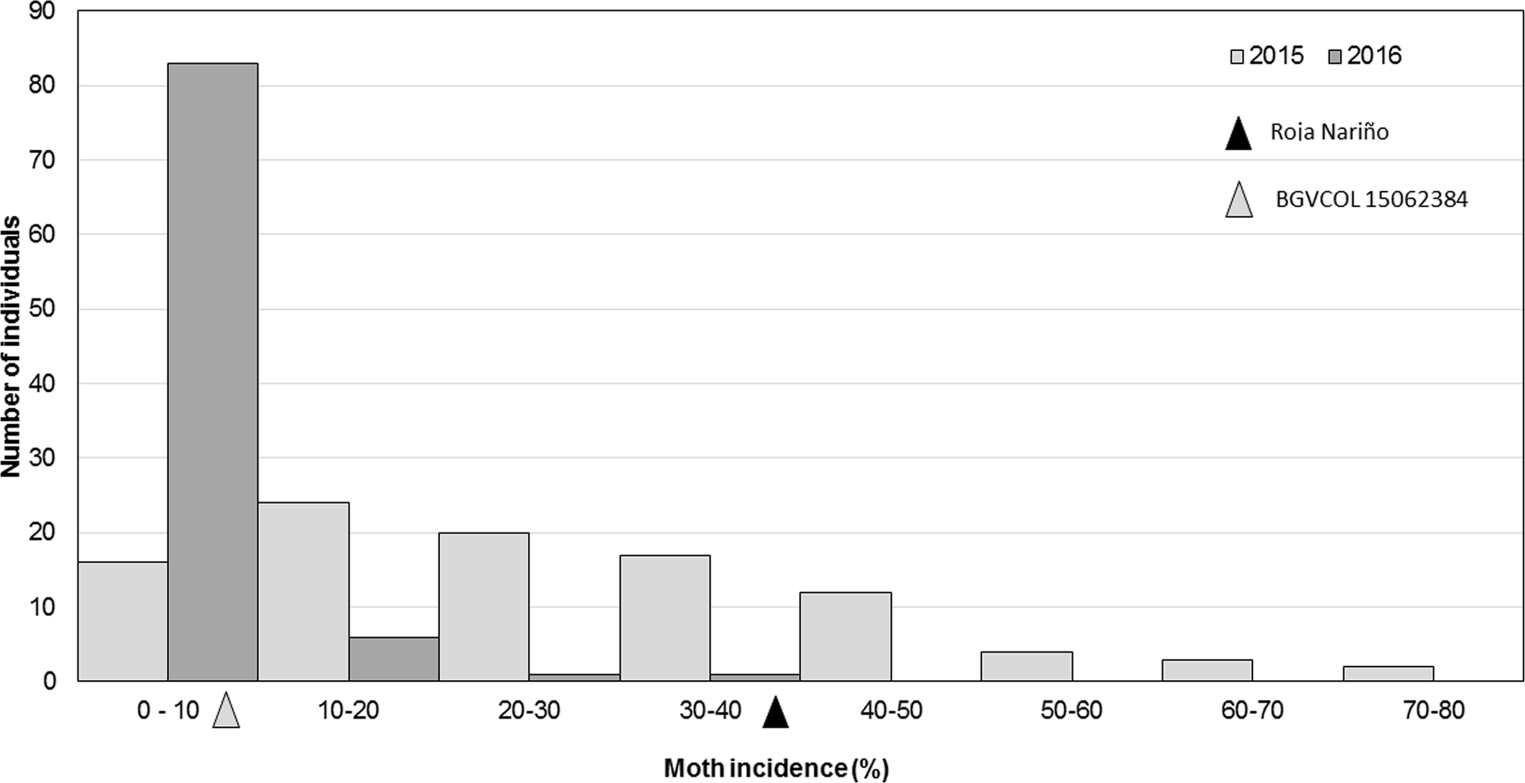
Incidence of *Tecia solanivora*. Histogram of incidence of number of individuals of *Tecia solanivora* under field conditions in two consecutive years (2015 and 2016). The black triangle indicates the phenotypic value of Roja Nariño and the grey one indicates the value of BGVCOL 15062384.

Similarly, the incidence of *T*. *solanivora* exhibited a differential pattern comparing years of assessment. In 2015, the average incidence was 73% due to low precipitation occurrence during flowering and senescence, allowing the moth to be optimally developed and increasing adult number (Fig 2). In contrast, in 2016 humidity reduced the adult moth population as well as its incidence with an average of 5.07% (Fig 3). As it was expected, the susceptible parent Roja Nariño potato showed an incidence of 20.98% and BGVCOL 15062384 showed a moderate resistance of 2.41% (Table 1). Interestingly, the population distribution during 2015 presented a biased and unimodal trend, indicating that few major genes are associated with moth resistance (Fig 3) [44]. Moreover, this contrasting response of *T*. *solanivora* incidence based on rainfall, relative humidity and temperature has been reported previously by Villanueva and Saldamando [45]. Additionally, in 2016 two genotypes with higher values compared to their susceptible parents were identified, and 20 genotypes showed lower degree of severity than their resistant parent (negative transgressive segregation), indicating a possible genetic overdominance of some loci.

**Fig 4:**
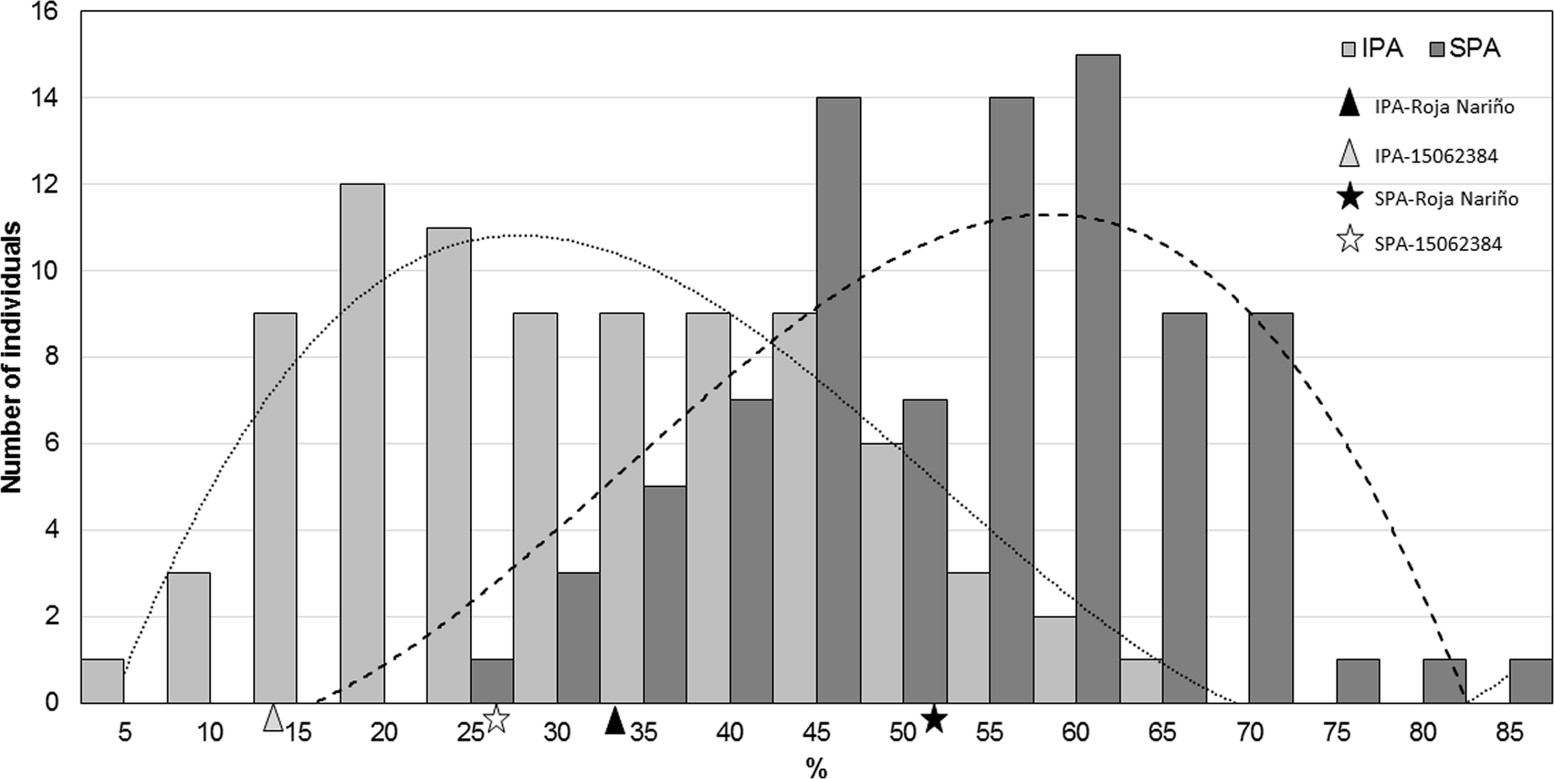
Incidence (IPA) and severity (SPA) of *Tecia solanivora* under storage conditions. Severity (SPA) and incidence (IPA) values are shown in dark gray and light-grey bars, respectively. The dash line indicates the SPA trend average and the dotted line indicates the IPA trend average. The black and gray symbols indicate the phenotypic value of Roja Nariño and BGVCOL 15062384. Triangles and stars indicate IPA and SPA respectively.

**Fig 5:**
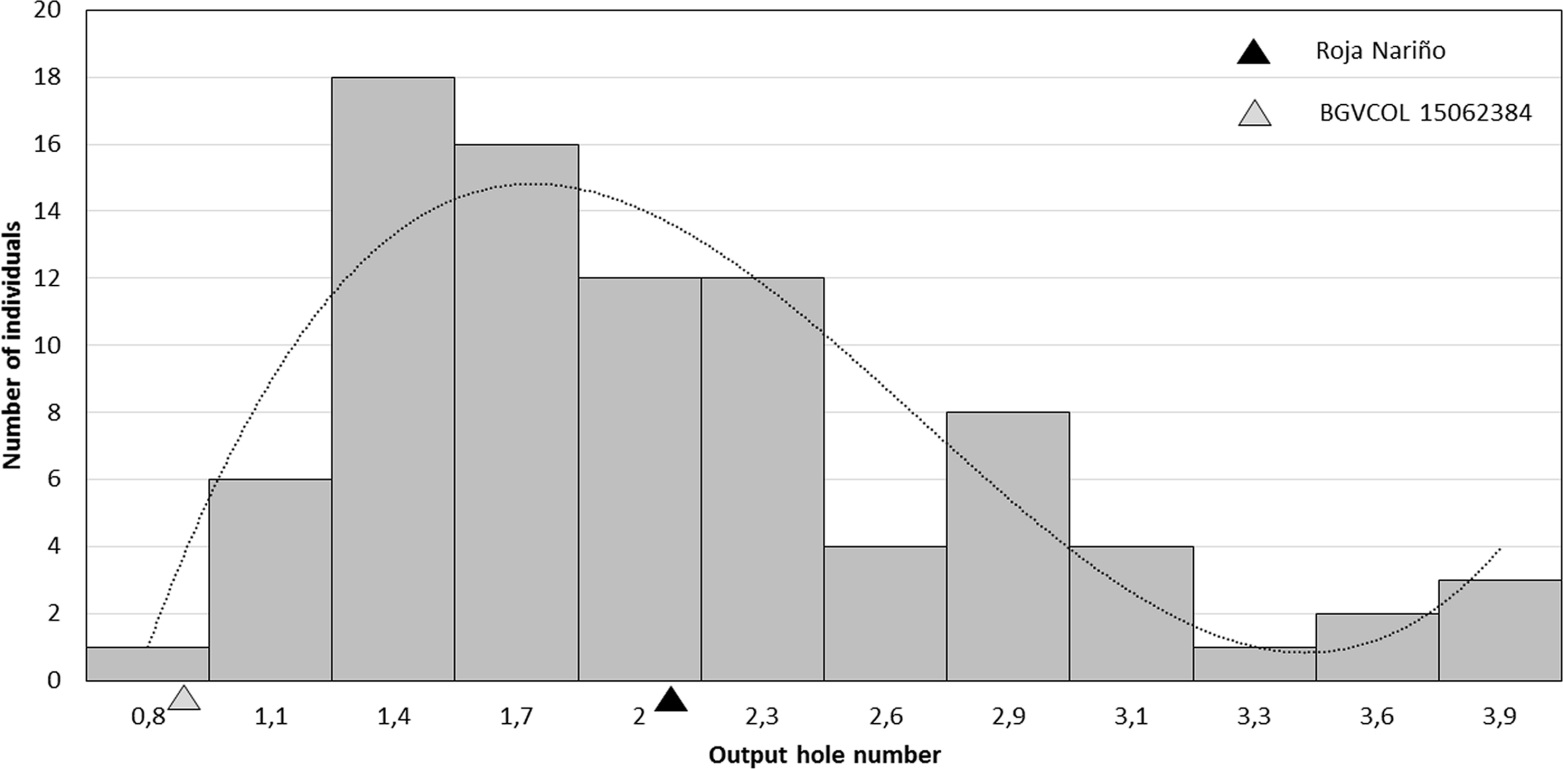
*Tecia solanivora* severity based on number of output holes (OPA). The x-axis shows the output holes number in each tuber, while on the y-axis shows number of individuals. The dotted line indicates the average trend. The black triangle indicates the phenotypic value of Roja Nariño and the grey one de value of BGVCOL 15062384.

In addition, the storage trial showed a higher damage compared to field conditions, where the moderate resistant parent BGVCOL 15062384 showed less incidence (15.78%), severity (24.71%) and output holes (0.97) compared with Roja Nariño that showed an incidence of 34.54%, a severity of 52.08% and 1.6 output holes. The F1 population showed a normal distribution for incidence, severity and number of output holes (Fig 4 and Fig 5). Additionally, we found transgressive genotypes for resistance and susceptibility supporting our field results. In average, the population showed incidence of 29.93%, severity of 51.58% and 1.92 output holes (Table 1). Similar results using the same methodology were found by Cadena *et al*. [46] that reported a range of severity from 3% to 90% with an average of 45.1%, and using a severity selection threshold of 25%. In contrast to the polygenic inheritance found in the storage trial, Ortiz *et al*. [47] reported a simple inheritance resistance from the parent *Solanum sparsipilum* (Bitter) Juz. & Bukasov to potato tuber moth (*Phthorimaea operculella*), evaluating 62 full-sibling families. In this difference, it is important to define the breeding strategies used (population size, selection pressure, and selection cycles) as polygenic traits are more complicated in terms of genetic gain [48].

### Genotyping

A total number of 12,808 SNPs from SolCAP potato array including SNPs 8,303 previously reported by Hamilton *et al*. [24], plus additional SNPs developed from candidate genes were used (Joseph Coombs, personal communication). Subsequently, 878 markers were discarded based on low allelic dosage quality. A total of 5,460 (45.8%) SNPs were polymorphic, 55 were incompatible with the parent allelic dosage, 967 showed double reduction, which in terms of tetrasomic inheritance, this is one of the biological causes generating more distortion in population segregation [49,50], and 1,954 presented 0 allele dosage for both parents (AAAA x BBBB). Finally, 1,606 (13%) markers were selected to be assigned to a chromosome; and of these, 291 markers were discarded as duplicates using a two point analysis (TPA), and 28 markers were found as outliers by multi-dimensional scaling (MDS), according to the methodology reported by Preedy and Hackett [32].

### Linkage mapping

A total number of 1,287 markers were mapped into 12 chromosomes according to the physical map (PGSC v4.03) [26]. More than half of the markers (791 markers) segregated in the 1:1 or simplex form (AAAB × AAAA, ABBB × BBBB), and the other informative markers segregated as double simplex (AAAB × AAAB, ABBB × ABBB) and duplex (AABB × AAAA, AABB × BBBB). These specific dosage configurations allow the detection of linkages between pairs of loci and estimation of recombination frequencies [22]. In general, markers showed a uniform distribution within each chromosome with a range of 74 markers on chromosome 12, and 197 on chromosome 1, with an average of 107 markers per chromosome (Table 2).

**Table 2:** Summary of the genetic map of the potato population RN × 2384.

Chromosome	Mapped markers	Length map (cM)	Interval density (cM)	Long Interval (cM)
1	197	108.3	0.55	7.31
2	95	70.2	0.74	7.18
3	108	88.08	0.82	6.08
4	131	82.88	0.63	7.03
5	90	70.96	0.79	10.7
6	113	76.15	0.67	5.40
7	91	91.09	1.00	15.04
8	88	63.9	0.73	4.30
9	102	83.9	0.82	7.34
10	99	96.51	0.97	9.87
11	99	66.74	0.67	4.26
12	74	87.74	1.19	9.40
**Total**	**1,287**	**986.4**		

Average density between markers across the genome was 0.77 cM with a minimum of 0.55 cM on chromosome 1 and a maximum of 1.19 cM on chromosome 12. The total map length was 968.4 cM with a mean of 82.2 cM per chromosome, a minimum length of 63.9 cM for chromosome 8 and a maximum of 108.3 cM for chromosome 1. Some intervals larger than 10 cM were found in the genetic map on chromosomes 5 and 7, with gaps of 10.7 cM and 15.04 cM, respectively.

Similar map length and density were reported by Hackett *et al*. [22] using a F1 population of 190 full-sibs and 1,301 SNPs, reporting a total map length of 1,087 cM with a density of 0.83 cM. Likewise, Massa *et al*. [14] showed a map length of 1,072 cM with a density of 0.54 cM using 1,972 SNPs. Recently, da Silva *et al*. [27] mapped 2,426 SNPs using 236 F1 individuals, reporting a map size of 1,052.6 cM with a density of 0.98 cM. Thus, our study showed a similar total map length and marker density compared with the results mentioned above, despite the relatively small population size (Table 2). However, when the number of progeny genotypes is around 100, the variances are highly overestimated; this can however be reduced by increasing the population size [51].

### *Phytophthora infestans* QTL identification

**Fig 6:**
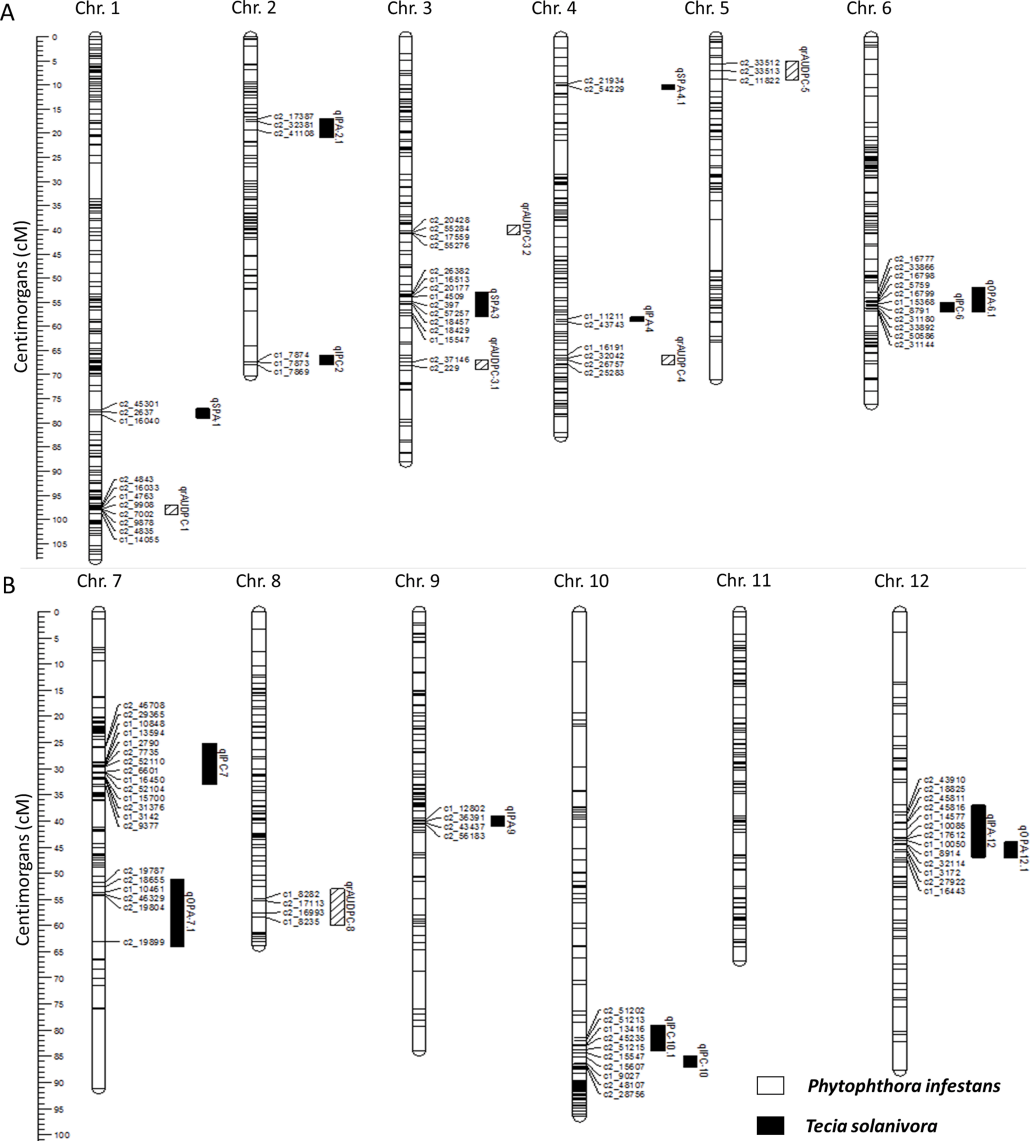
Linkage map and QTLs for *Tecia solanivora* and *Phytophthora infestans*. The position of the marker in centimorgans is shown in the y-axis. Black bars show *T*. *solanivora* QTL locations, meanwhile white bars show *P*. *infestans* QTL locations.

Six QTLs were identified for resistance to *P*. *infestans*, more specifically, four QTLs for resistance (qrAUDPC-1, qrAUDPC-3.2, qrAUDPC-5 and qrAUDPC-8) and two for susceptibility (qrAUDPC-3.1 and qrAUDPC-4) (Table 3 and Fig 6). These QTLs were not stable from one year to the next mainly due to the climatic variability occurred in 2015 and 2016 (S1 Fig), and minor QTLs are highly affected by the environment. The percentage of explained phenotypic variance ranged from 3.93% to 7.17% with a log of the odds (LOD) score ranging from 2.86 to 3.43 on chromosomes 3 and 1, respectively, indicating that they are minor QTLs. According to the Beavis effect, this could be because the RN × 2384 population only had 94 genotypes, and this small population size may reduce the LOD score of major QTLs; on the other side, maybe it cannot detect QTLs of smaller effects reducing the number of QTLs detected [51]. Vales *et al*. [52] have studied this effect in wheat rust resistance identifying major QTLs but missing those of small effects in a relatively small population. This is an important issue especially for partial resistance to LB, where the QTLs reported showed small effects [20,53]. In contrast, QTLs related to *Globodera pallida* resistance showed a significantly higher effect [53].

**Table 3:** QTLs for resistance to *Phytophthora infestans* found in the RN × 2384 population.

QTL	Chr	Year	Marker	Position cM	Position Mb^a^	LOD	LOD 90% ^b^	*R*^2^	Gene function
**qrAUDPC-3**	3	2015	c2_229	68	57.97	2.9	2.73	3.9	Auxin response factor 2
**qrAUDPC-8**	8	2015	c2_16993	58	54.8	3.5	2.56	5.2	BHLH transcription factor JAF13
**qrAUDPC-5**	5	2016	c2_11822	7	4.05	2.9	2.48	4.2	E3 ubiquitin ligase PUB14
**qrAUDPC-4**	4	2016	c1_16191	67	65.9	2.5	2.47	5.4	Receptor protein kinase CLAVATA1
**qrAUDPC-3.1**	3	2016	c2_17559	40	44.14	3.1	2.64	5.6	ABC-type Co2+ transport system
**qrAUDPC-1**	1	2016	c2_9878	98	81.5	3.4	3.08	7.2	Unknown function

^a^ Location on the physical map (Mb) according to PGSC v4.03. LOD score.

^b^ LOD score at a threshold calculated at 90% with 1,000 permutations; Chr: chromosome.

Furthermore, of the QTLs related to LB resistance, four have previously been reported in the literature. Particularly, according to the synteny analysis performed by Mosquera *et al* [1], the QTL qrAUDPC-1 found at 81.5 Mb on chromosome 1 is placed 0.46 Mb from the QTL dd1b reported in tomato [54]. Additionally, the region comprised by qrAUDPC-1 shows a gene that codes for a zinc finger protein that has multiple functions in the regulation of several resistance mechanisms to biotic and abiotic factors. Particularly, zinc finger domains are present in the resistance proteins accompanied by nucleotide-binding site leucine-rich repeat (NBS-LRR) proteins [55], which have been shown to lead to programmed cell death through the hypersensitive response [18]. Additionally, the locus found is related to nodulin proteins, which shows sugar transporter domains called Sweets involved in plant susceptibility, senescence, and glucose uptake in *Arabidopsis* roots [56].

Moreover, the QTL qrAUDPC-3.1 found on chromosome 3 is collinear with the QTL GP25 reported by several authors [57–61]. This locus showed a gene related to a component of the ABC transporter membrane, which is involved in resistance to bacteria, fungi and insects [62–63]. One type of ABC transporter is the NtABCG5/NtPDR5, and its presence in the membrane is directly related to plant resistance to herbivore attack between the hornworm (*Manduca sexta* L.) and tobacco (*Nicotiana tabacum* L.) involving methyl jasmonate signaling [64].

Similarly, the QTL qrAUDPC-5 found at 4.05 Mb in chromosome 5 is closely related to QTL GP21 [57,58,65,66]. The same loci have been associated to maturity [1,14,20,67], and a StCDF1 gene (PGSC0003DMG400018408), which is a maturity gene in potato plants, has been found in this region. The QTL qrAUDPC-4 was related to susceptibility and was located at position 65.9 Mb with a syntenic relation to the QTL TG345 reported by Brouwer *et al*. [54] in tomato. In addition, individuals with this allele from the susceptible parent in locus c1_16191, have on average a higher disease severity (0.071 rAUDPC). This mapped locus is related to the kinase protein receptor of the cytoplasmic membrane CLAVATA 1, which has extracellular domain leucine-rich repeats (LRRs), and a functional domain of kinases rich in serine-threonine, which acts in signal transduction [68]. This type of domain has been shown to have the function of recognizing pathogens by signaling cascades, and the subsequent activation of defense mechanisms in plants [69–70].

On the other hand, qrAUDPC-3 and qrAUDPC-8 have not been reported previously. The first one was located in the 57.97 Mb position and individuals with this allele from the susceptible parent in locus c2_229 has an average higher disease severity (0.015 rAUDPC). Moreover, this locus involved a transcription factor Arf2 related to leaf senescence, mediated by oxidative stress in *Arabidopsis* [71], and is related to the signaling of the gibberellin and brassinosteroid pathways in the plant-pathogen interaction [72,73], Specifically, wheat plants with this silenced gene showed a moderate but significant reduction in the severity of wheat blight (FHB) symptoms caused by *Fusarium culmorun* [74], mainly due to auxin signaling that helps colonization in a plant-pathogen interaction [75–77]. The QTL qrAUDPC-8 was located at the 54.8 Mb position and individuals with this allele of the susceptible parent at locus c2_16993 have on average a lower disease severity (-0.018 rAUDPC). This marker is related to a gene that codes for a transcription factor helix-loop-helix (bHLH) JAF13 involved in flavonoid biosynthesis in *Petunia x hybrida* [78].

These results should be validated in other environments and with another genetic background to corroborate the QTL effect on resistance due to non-additive effects and environmental interaction, as these play an important role for quantitative resistance response to *P*. *infestans* [79]. It is also important to note that QTL-associated genes identified in this study (Table 3) are known to be important for plant defense and disease resistance (i.e. auxin-mediated defensive mechanisms, defense-related transcription factors, protein degradation, cell signaling and ABC-associated transport).

### *Tecia solanivora* QTL identification

The QTLs associated with resistance or susceptibility to *T*. *solanivora* found in this study are so far, the first reported in the literature. These QTLs are different from those identified for *P*. *infestans* in this evaluation. A total of fifteen QTLs for *Tecia solanivora* were identified, five QTLs for the field phenotypic trial (IPC), including three QTLs in 2015 and two in 2016. Under storage conditions, ten QTLs were identified including four related to moth incidence (IPA), three for moth severity (SPA) and three for moth output holes (OPC) (Table 4 and Fig 6). In summary, seven QTLs were found related to resistance, and eight to susceptibility. Interestingly, despite the precipitation difference occurred between years (S1 Fig) some QTLs were stable such as qIPC-6 in 2015 and qOPA 6.1 in 2017, and qIPC-10 in 2015 and qIPC10.1 in 2016.

**Table 4:** QTLs identified for resistance to *Tecia solanivora* in the RN x 2384 population.

QTL	Chr	Year	Marker	Position (cM)	Position (Mb)	LOD score	LOD Threshold 90%	*R*^2^	Gene function
**qIPC-2**	2	2015	c1_7874	67	46.19	2.83	2.79	4.8	Granule-bound starch synthase 2
**qIPC-6**	6	2015	c2_33892	56	49.67	2.77	2.59	3.7	Cytochrome c1-1. heme protein
**qIPC-10**	10	2015	c1_13416	86	55.89	2.38	2.35	3.9	Transcription initiation factor IIB-2
**qIPC-7**	7	2016	c2_52110	29	9.12	4.15	2.55	11.4	Auxin response factor
**qIPC-10.1**	10	2016	c2_48107	82	58.4	2.77	2.36	5.7	F-box family protein
**qIPA-4**	4	2017	c1_11211	58	62.25	2.86	2.77	3.8	Regulator of Vps4 activity in the MVB pathway
**qIPA-2.1**	2	2017	c2_32381	51	22.39	4.49	2.99	12.9	Conserved gene of unknown function
**qIPA-9**	9	2017	c2_43437.	40	47.65	2.91	2.81	4.8	Cyclin-dependent protein kinase
**qIPA-12**	12	2017	c1_16443	41	10.04	3.55	2.67	8.3	Enhancer of ag-4 1
**qSPA-3**	3	2017	c2_20177	54	48.94	3.68	2.64	8.6	AGO1-2
**qSPA-4.1**	4	2017	c2_21934	10	4.60	2.63	2.73	5.2	CD2 antigen cytoplasmic tail-binding protein 2
**qOPA-7.1**	7	2017	c2_10461	54	32.40	3.61	2.47	10.3	Vacuolar protein sorting protein
**qOPA-6.1**	6	2017	c2_31144	57	48.87	2.46	2.17	4.8	Unknown function
**qOPA-12.1**	12	2017	c1_3172	45	29.22	2.33	2.24	4.3	Cleavage stimulation factor 50 kDa subunit

Chromosome (Chr); evaluation year (Year); Marker position (Position Mb) on the physical map according to PGSC v4.03; LOD score at a threshold calculated at 90% with 1,000 permutations (LOD Threshold 90%); % of explained variance (*R*^*2*^).

Among the four QTLs found for resistance in the field (qIPC-10, qIPC-6, qIPC-7 and qIPC-2), the QTL qIPC-2 at 67 cM in chromosome 2 contributed in the additive effect to the quantitative resistance with an incidence percentage of -13.05%. This means that within this population, individuals with the resistant potato allele at locus c1_7874 had on average a lower pest incidence. On the same chromosome at 52 cM the susceptibility QTL qIPA-2.1 was observed where the highest LOD score (12.99) and the highest explained variance (4.49%) were found. The same locus (PGSC0003DMB000000085) was found by Manrique-Carpintero *et al*. [80] using association mapping, identifying a gene related to Leptin type II, a type of hormone related to the steroidal glycoalkaloid synthesis pathway(SGAs). This compound has been found in high concentrations in *Solanum chacoense* Bitter, which is known as a resistance source to the Colorado potato beetle (*Leptinotarsa decemlineata*) [81].

Two QTLs were found on chromosome 7. The first QTL is qIPC-7 at 29 cM with an additive effect on the incidence of -4.49%, and this locus is related to a gene that codes for an auxin response factor Arf19, which functions as a repressor or transcriptional activator of 3-indoleacetic acid [82]. Furthermore, Xu *et al*. [83] have shown that this family of auxin response factors plays an important role in signaling abiotic stress events and plant development in tea plants (*Camellia sinensis* (L.) Kuntze). The second QTL found on chromosome 7 has an additive effect of -1.05 outflow holes. This locus is related to a subunit of the multiprotein proteasome complex RPN1, which has endoprotease activity, and has been found in *Arabidopsis* plants, where the accumulation of RPN1 is positively affected by salicylic acid (SA). This in turn relates to the basal immunity response to various pathogens, but when this subunit mutates, the SA level decreases facilitating the infection of the powdery mildew caused by *Golovinomyces cichoracearum* [84].

The QTLs qIPC-10 and qIPC-10.1 placed at 55.89 Mb and 58.4 Mb identified during 2015 and 2016, respectively, showed a stable QTL region. The genotypes with this allele from the resistant parent has an incidence damage percentage of -6.53% and -8.94%, respectively. The first locus is related to the transcription factor TFIID, which provides the recognition of the promoter and has a catalytic function for the synthesis of mRNA in eukaryotic organisms [85]. Another gene reported in this region codes for the enzyme glucosyltransferase, which catalyzes the glucose residues transfer for polysaccharide synthesis, and is related to the synthesis route of abscisic acid (ABA). In addition, in mutant *Arabidopsis* plants a relationship of this gene with tolerance to water stress [86] was demonstrated. Likewise, QTL qIPC-10.1 is related to a type of F-box protein, which contributes to resistance to phytopathogenic bacteria; this protein is involved in the regulation of hormones such as ABA, which is the central mediator of signaling responses to different stresses and plant defense by other phytohormones [87].

Similarly, two susceptibility QTLs were found on chromosome 6. The QTL qIPC-6 for field incidence was located at the same position as qOPA-6.1 that was found under storage conditions. This locus is 1.8 Mb from superscaffold PGSC0003DMB000000578 (cytochrome P450 71D7) found by Manrique-Carpintero *et al*. [80], related to putative SGAs genes, specifically α-solanine and α-chaconine in the resistance to the Colorado potato beatle (*L*. *decemlineata)*. These genes were found with catalytic characteristics like *GAME7* and *GAME8* genes in tomato, involved in the transformation of cholesterol to tomatidenol in α-tomatine synthesis [88].

In addition, in the evaluation under storage conditions, two QTL related to susceptibility were found in the same position in a range of 37 to 47 cM of chromosome 12 (qIPA-12 and qIPA-12.1). The qSPA-3 explains 8.63% of the variance and is related to the Argonaute gene AGO1, where its mutant allele is related to susceptibility to the cucurbit mosaic virus (CMV) in *Arabidopsis* [89]. Furthermore, this family of genes are related to the transcriptional silencing pathways of genes through RNA interference (RNAi) in insects, showing death or non-viability in the silkworm *Bombyx mori* via ingestion [90]; however, it has been proposed as an alternative for integrated pest management [91](16). The marker found in qSPA-1 is related to the gene glutaredoxin ATGRXS13 involved in the infection of *Botrytis cinerea* in *Arabidopsis*, regulating jasmonic acid expression [92].

Finally, QTLs qIPA-4 and qSPA-4.1 located at position 58 and 10 cM, respectively, were found on chromosome 4. The QTL qIPA-4 shows an additive effect on resistance of -6.76% incidence, while qSPA-4.1 presents an effect of -8.94% on severity. QTL qSPA-4.1 (c2_21934, chr04: 4606550) is colocalized with the resistance QTL PCN_res for *Globodera pallida*, which was reported between markers c1_16358 (chr04: 3240076) and c2_21847 (chr04: 5030164), with a LOD score of 16.6 and an explained variance of 29.8% [22,93].

Nonetheless, polygenic resistance to *T*. *solanivora* involves antibiosis and/or antixenosis mechanisms, reducing the rate of pest growth [94]. This resistance is based on molecule signaling associated with metabolic pathways of basal plant defense as primary metabolites (citric acid, cysteine and aromatic amino acids) and secondary ones such as jasmonic acid, salicylic acid, abscisic acid, ethylene, cytokinins and glycoalkaloids [95–98]. Interestingly, QTLs qIPA2.1, qIPC-6, and qOPA-6.1 reported here are involved on some on these pathways. However, these substances are toxic to humans within a maximum threshold of 200 mg per fresh weight consumed, according to studies with commercial cultivars [99].

Additionally, the attack of *T*. *solanivora* activates systemic defense mechanisms in the plant, increasing the transcription of the gene Lipoxygenase 3 (*Lox 3*) in leaves. This contributes to the synthesis of signaling molecules associated with plant defense in jasmonic acid and ethylene routes; hydroxycinnamoyl-CoA quinate hydroxycinnamoyl transferase (HQT) and 3-hydroxy-3-methylglutaryl CoA reductase I (HMGR1) genes are involved in the biosynthesis of chlorogenic acid and SGAs, respectively [100,101]. Putative genes involved in these SGAs were found related to QTLs qIPA2.1, qIPC-6, and qOPA-6.1. On the other hand, further phenotyping efforts should be focused on volatile compounds. For instance, 6-methyl-5hepten-2-one (sulcatone) found in tubers has been reported to reduce the attraction to *T*. *solanivora*, while others such as methyl phenylacetate (MPA) emitted by flowers and in tubers increases the attraction of *T*. *solanivora* adults [102], allowing an effective oviposition in plant roots [103](18). In addition, the attack of lepidopteran insects such as *Spodoptera exigua* and *Spodoptera frugiperda* do not have a systemic resistance effect against the attack of *T*. *solanivora* [101]. Consequently, it is extremely likely that the results in this study are based on genetic effects inherited from the parent that carries the resistance, instead of a systemic resistance acquired by the attack of another insect. However, further studies must be carried out to validate these results and to understand the biochemical and metabolic pathways involved on the genetic response to pest resistance. Additionally, the resistance and susceptibility of the QTLs found in this study will be an important information to look at for function and gene pathway validation using silencing or knock-out approaches such as virus induced gene silencing (VIGS) in resistance QTLs [104], and genome editing using the CRISPR/Cas9 system in susceptible QTLs [105,106].

### Conclusions

This study used the linkage mapping approach to identify QTLs for resistance to *P*. *infestans* and *T*. *solanivora*. QTLs linked to *P*. *infestans* were identified and mapped, four of them very close to QTLs reported previously; meanwhile fifteen QTLs for *T*. *solanivora* were identified for the first time. These results are a valuable tool for development of cultivars with partial resistance to *P*. *infestans* and *T*. *solanivora*. However, these QTLs are going to be validated in different environments and genetic backgrounds, in order to be routinely used on marker-assisted selection in a potato-breeding program in Colombia.

## Supporting information

S1 Table Phenotypic values of the F1 progeny. (XLSX)

S1 Fig Weekly and total precipitation ocurred in two crop cycles during 2015 and 2016. (A) Weekly precipitation. (B) Total precipitation.
(TIF)
