# Supplementary figures and images for: QTL analysis reveals quantitative resistant loci for Phytophthora infestans and Tecia solanivora in tetraploid potato (Solanum tuberosum L.)
Source: PLoS One. 2018 Jul 6;13(7):e0199716. doi: 10.1371/journal.pone.0199716 (PMC6034811; doi:10.1371/journal.pone.0199716)

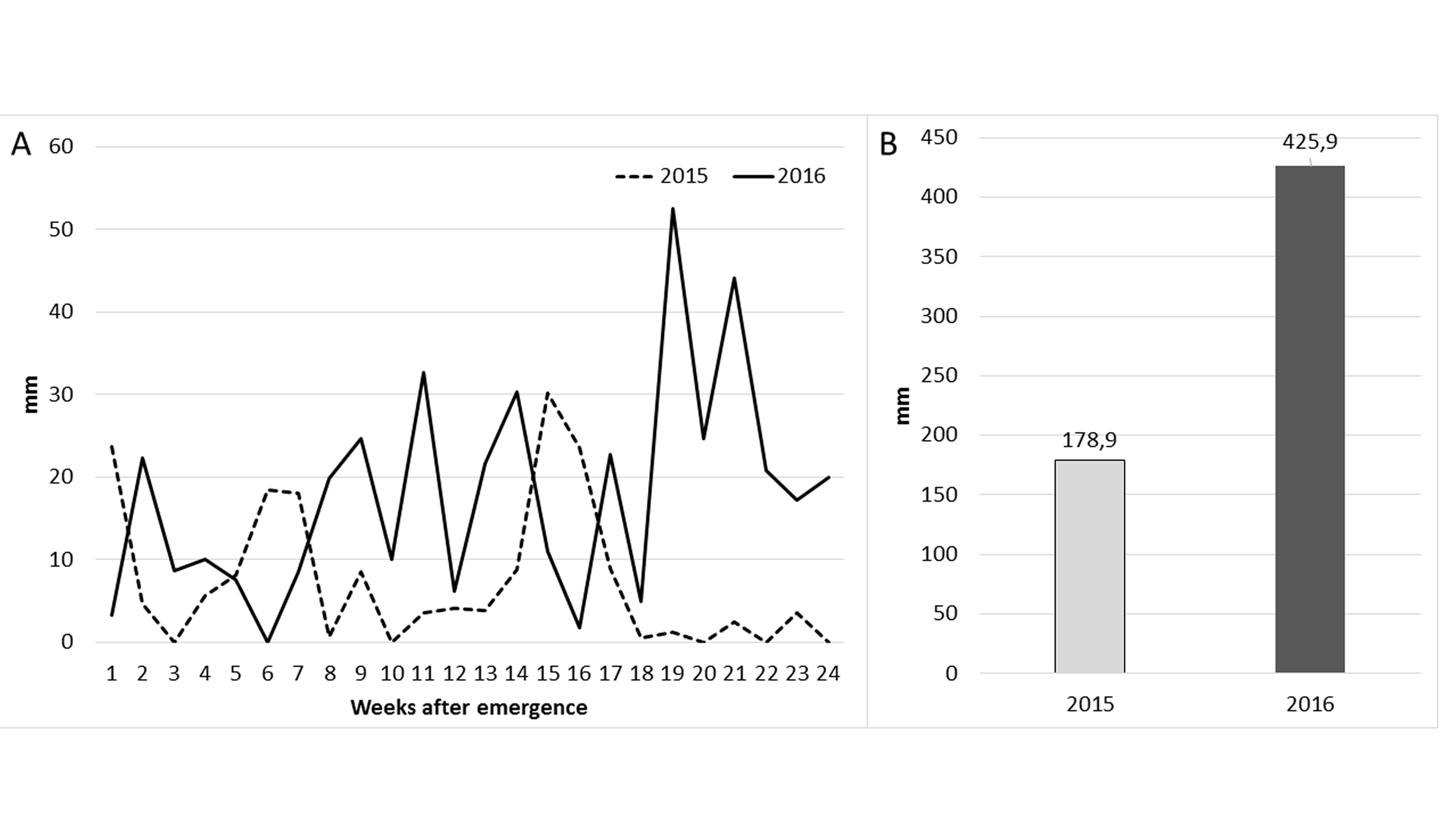

Supplement: S1 Fig — (A) Weekly precipitation. (B) Total precipitation. (TIF) [file pone.0199716.s002.tif]
